# Ambient temperature and sunshine hours are not associated with type 1 diabetes incidence in subtropical Taiwan: a nationwide time-series analysis

**DOI:** 10.1016/j.clinsp.2026.101050

**Published:** 2026-07-08

**Authors:** Ya-Hui Chang, Hon-Ping Ma, Hua-Fen Chen, Ping-Ling Chen, I-Lin Hsu, Chung-Yi Li

**Affiliations:** a School of Public Health, College of Public Health, Taipei Medical University, Taipei, Taiwan; b Graduate Institute of Injury Prevention and Control, College of Public Health, Taipei Medical University, Taipei, Taiwan; c Emergency Department, Shuang Ho Hospital, Taipei Medical University, New Taipei City, Taiwan; d Department of Emergency Medicine, School of Medicine, College of Medicine, Taipei Medical University, Taipei, Taiwan; e Department of Endocrinology, Far Eastern Memorial Hospital, New Taipei City, Taiwan; f Department of Medicine, College of Medicine, Fu Jen Catholic University, New Taipei City, Taiwan; g Department of Public Health, College of Medicine, Fu Jen Catholic University, New Taipei City, Taiwan; h Division of Surgery, National Cheng Kung University Hospital, College of Medicine, National Cheng Kung University, Tainan, Taiwan; i The Cross College Elite Program, National Cheng Kung University, Tainan, Taiwan; j Department of Public Health, College of Medicine, National Cheng Kung University, Tainan, Taiwan; k Department of Public Health, College of Public Health, China Medical University, Taichung, Taiwan; l Department of Healthcare Administration, College of Medical and Health Science, Asia University, Taichung, Taiwan

**Keywords:** Type 1 diabetes, Incidence, Ambient temperature, Sunshine hours, Subtropical climate, Ecological study, Time-series analysis, Taiwan

## Abstract

Seasonal patterns in Type 1 Diabetes (T1D) suggest environmental factors play a role, with colder months linked to higher incidence rates. While low temperatures are a known risk factor, the impact of exposure to shorter sunshine hours is less clear. This study investigated the associations between ambient temperature, sunshine hours, and the incidence of T1D in Taiwan. This cohort study analyzed 3,146 T1D cases aged < 35-years from Taiwan’s National Health Insurance data (2015‒2022). The relationships between the weekly number of T1D incident cases, mean daily temperature in a week, and total sunshine hours in a week were evaluated using a distributed lag nonlinear model. When both meteorological factors were considered separately or simultaneously, significantly reduced RRs (0.86‒0.95) of T1D incidence were observed across the temperature range of 17‒19 °C, referenced to 20 °C, but no significant associations with T1D incidence were found for weekly sunshine hours within the observed range of 12–61 h. This study shows that neither lower temperature nor shorter sunshine exposure was significantly associated with increased T1D incidence in Taiwan, potentially due to the region’s subtropical climate. Interpretations of the observed reduced risk within the temperature range of 17–19 °C should be made with caution, as this finding may be influenced by residual confounding from seasonally correlated factors, such as viral circulation, behavioral patterns, and indoor environmental conditions.

## Introduction

Numerous studies have documented a seasonal clustering of Type 1 Diabetes (T1D), with higher incidence rates observed during colder seasons in both the Northern and Southern Hemispheres.^1^, ^2^, ^3^, ^4^, ^5^ This seasonal variation suggests a link between environmental factors and T1D onset. The increase in diagnoses during autumn and winter may be attributed to heightened viral activity during these seasons, with geographical differences potentially reflecting the impact of atmospheric temperature.^6^

Sunshine exposure is another environmental factor considered in the seasonal variation of T1D. While the association between low temperatures and increased T1D risk is well-supported, evidence linking exposure to shorter sunshine hours to higher T1D incidence is less conclusive.^4^^,^^7^^,^^8^ An early Swedish study suggested that both low temperatures and short daylight hours contribute to the seasonal variation in T1D,^7^ but later research emphasized that temperature, rather than sunlight, is the primary driver of this pattern.^8^ For example, Patterson et al. found that deviations in monthly T1D diagnoses across 23 European centers were significantly associated with temperature fluctuations but not with sunshine hours.^4^

Nonetheless, these studies were conducted predominantly in cold, temperate regions, focusing mainly on Caucasian and pediatric populations. The generalizability of these findings to other regions or adult populations is uncertain. To address these gaps, the authors conducted a population-based cohort study in Taiwan, a subtropical country, analyzing all incident cases of T1D from 2015 to 2022 to further explore the links between ambient temperature, sunshine hours, and T1D incidence.

## Materials and methods

The study proposal was approved by the Institutional Review Board of National Cheng Kung University Hospital (n°B-ER-112–034). Informed consent was waived due to the use of anonymous personal identification numbers. Access to the research data was approved by the Health and Welfare Data Science Center (HWDSC). To guard the data, the data management and statistical analyses involved in this study were conducted on-site at HWDSC.

### Data source

This study utilized medical claim data from the National Health Insurance (NHI) program in Taiwan from 2015 to 2022. Patient visit dates, treatment medications, and patient characteristics were retrieved from both outpatient and inpatient medical claims. Ambient temperature data from all 344 meteorological stations across Taiwan from 2015 to 2022 were obtained from the Central Weather Administration. Among all stations, there are only 29 recorded sunshine hours data points. The authors excluded data from two stations located in high mountain uninhabited areas.

### Study design and sample

A retrospective study design was used. Daily data on the incident cases of T1D diagnoses as well as ambient temperature and sunshine hours, were retrieved. Between 2015 and 2022, a total of 3146 incident cases of T1D aged ≤ 35-years were identified over 2922-days in four geographic regions (i.e., North, Central, South, and East) of Taiwan. To obtain a more reliable estimation, the authors further aggregated cases diagnosed in the same week in the analysis, yielding a total of 420-weeks of observations. The number of cases < 35-years per week ranged from a minimum of 2 to a maximum of 37, with a median of 8 cases/week and a mean of 9 cases per week during the study period. The ecological design aimed to capture population-level temporal variation rather than individual exposure.

Many population-based incidence studies and registers report T1D incidence using 0‒14 years (childhood) and 15‒34 years (young adult) strata, making < 35 a pragmatic cutoff that matches established surveillance practice and facilitates comparability across settings. For example, UK incidence trends have been reported explicitly for 0‒14 and 15‒34 year-olds.^9^ Likewise, Swedish validated research registers record incident T1D for 0‒14 and 15‒34 age groups (childhood registry since 1977; young-adult registry since 1983).^10^

### Identification of incident type 1 diabetes

The incident cases of T1D were retrieved from the NHI claim data based on the diagnostic codes, which were further confirmed by a record in the Catastrophic Illness Database (CID). However, individuals already diagnosed with T1D between 2012 and 2014 were excluded. Incident cases were determined based on the first diagnosis date with a CID record between 2015 and 2022, using ICD-9-CM codes 250.x1, 250.x3, or ICD-10-CM code E10.^11^ The diagnosis of T1D was confirmed using the CID. Reporting T1D to the NHI review board required a physician’s diagnosis certificate and relevant medical records, including examination results, fasting or glucagon-simulated C-peptide levels, anti-GAD antibody levels, and a history of diabetes ketoacidosis. The T1D diagnosis in the CID has previously been used to report the incidence of T1D in Taiwan, with a positive predictive rate of 98.3%.^11^^,^^12^

### Meteorological parameters

Although Taiwan is in the sub-tropical region, it also shows seasonal variations in ambient temperature and sunshine hours, with warmer temperatures and more sunshine in the summer months and cooler, less sunny conditions in the winter. For example, in 2022, the highest average weekly temperature was recorded in July, reaching up to 34.3 °C with minimum temperatures around 26.3 °C. On the other hand, the lowest average weekly temperature was observed in January, with an average high of 19.1 °C and a low of 13.9 °C. Sunshine hours also varied significantly. August had the most sunshine, averaging around 6.1 h of sunshine per day and 44.6 h per week. February had the least amount of sunshine, with an average of 2.5 h and 26.2 h of sunshine per day and week, respectively.^13^

All meteorological stations in Taiwan record hourly temperature data and daily sunshine hours. For this study, which used weekly data as the analytical unit, the authors first calculated the daily mean temperature and then averaged these values over seven days to represent the weekly mean daily temperature. Similarly, the authors summed up the recorded daily sunshine hours for the entire week to determine the total sunshine hours per week.

Weekly mean temperature and total sunshine hours were computed using the averages across all available stations in each of the four geographic regions (i.e., North, Central, South, and East) of Taiwan to represent exposure in specific regions. The T1D cases from the same geographic region shared the same exposure. No spatial interpolation or population-weighting was used in exposure assessment.

### Statistical analysis

A Distributed Lag Nonlinear Model (DLNM) was employed to examine the relationships between ambient temperature, sunshine hours, and the incidence of T1D.^14^ Initially, weekly mean daily temperature and total sunshine hours were analyzed separately in individual models. Subsequently, both variables were included in the same model to assess their independent associations with T1D risk. All models were adjusted for age at diagnosis, gender, season, calendar year, and month of diagnosis. Seasons were defined as spring (March to May), summer (June to August), fall (September to November), and winter (December to February).^15^

In this study, the dependent variable in the DLNM model was the weekly count of T1D incident cases, while the primary independent variables were the weekly mean daily temperature and total sunshine hours in a week. Since the outcome variable consists of count data, the authors applied a Poisson distribution, denoting the number of diagnoses in week *t* as Y*t*. However, because the Poisson distribution assumes that the mean and variance are equal, which may not always hold, the authors addressed potential over-dispersion by calculating an over-dispersion parameter (Φ) and using quasi-likelihood estimation. The model is expressed as E(Yt) = ϕVar(Yt), where ϕ > 1 indicates over-dispersion and ϕ < 1 indicates under-dispersion.^16^ The estimated dispersion parameter was φ = 1.13, indicating mild overdispersion. Given the relatively small degree of overdispersion, the quasi-Poisson model was considered an appropriate and parsimonious choice.

The reference values of 20 °C and 40 sunshine hours per week correspond to the respective median national values during the study period, allowing effect estimates to be interpreted relative to typical climatic conditions in Taiwan. Natural cubic spline specifications with 2‒5 degrees of freedom were compared, with AIC values of 2073.364, 2080.820, 2103.805, and 2092.083, respectively. Although the lowest AIC was observed for the 2-df model, the authors selected 3 degrees of freedom for the main model because it allowed slightly greater flexibility in modeling the exposure-response relationship.^14^ The model was implemented using the “*dlnm*” and “*spline*” packages in R software (version 4.4.1). An α-level was set at 0.05 to indicate statistical significance.

## Results

**Fig. 1:**
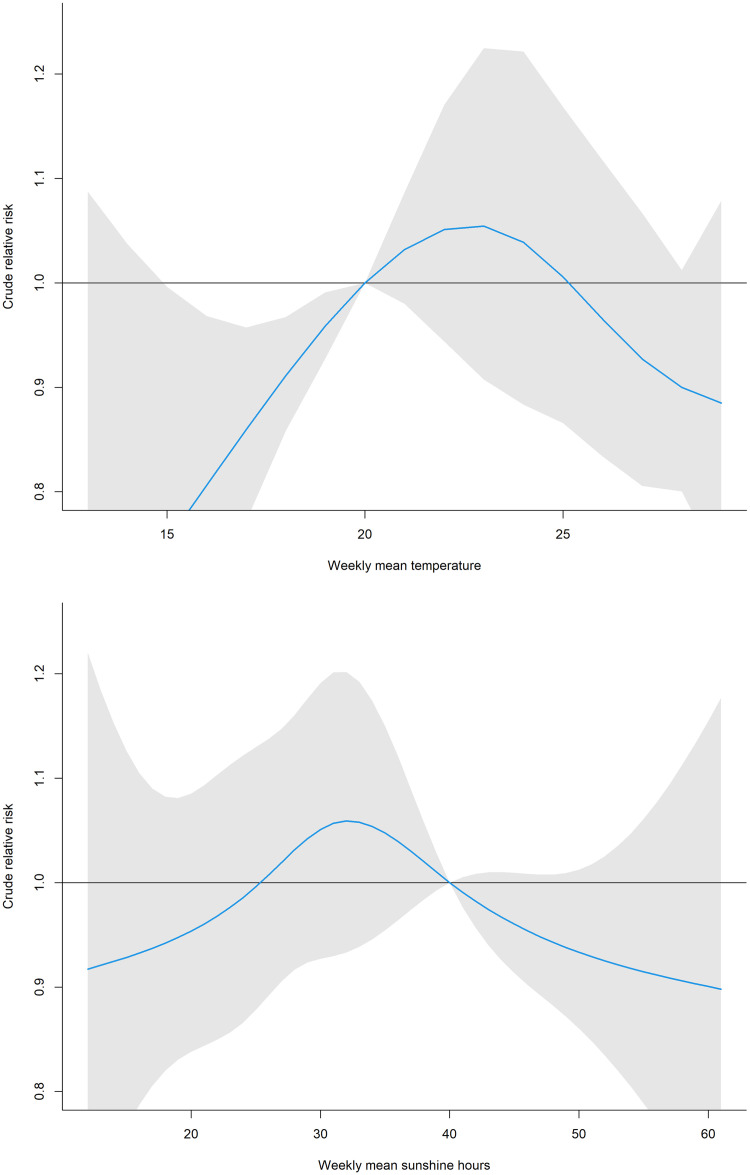
Relative risk of type 1 diabetes in association with exposures to weekly mean daily temperature (*upper*) and total sunshine hours per week (*lower*), with both factors assessed separately (n = 3146). Caption: Association between weekly mean daily temperature and the incidence of type 1 diabetes among individuals aged < 35-years in Taiwan, 2015‒2022. The solid line depicts the estimated Relative Risk (RR) based on a distributed lag nonlinear model, with a median of 20 °C set as the reference temperature. The shaded band between the dotted lines represents the 95% Confidence Interval (95% CI). The model adjusts for age, sex, region, season, calendar year, and month of diagnosis. Compared with the reference temperature (20 °C), the lowest and highest RRs (95% CI) for type 1 diabetes were 0.65 (0.39‒1.09) at 13 °C and 1.04 (0.89‒1.25) at 23 °C, respectively. Significantly lower RRs were observed within the temperature range of 17‒20 °C. In contrast, no significant association was identified between weekly total sunshine hours and type 1 diabetes across the range of 11‒61 hours.

**Fig. 2:**
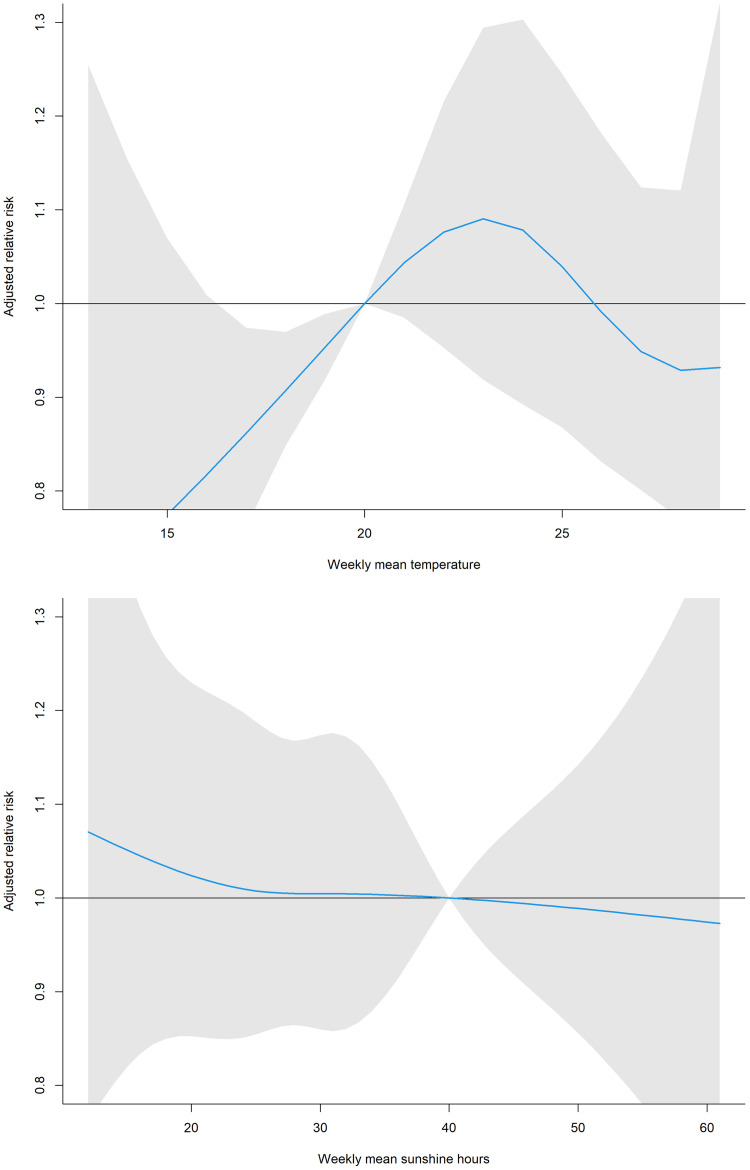
Relative risk of type 1 diabetes in association with exposures to weekly mean daily temperature (*upper*) and total sunshine hours per week (*lower*), with both factors assessed simultaneously to account for their independent effect (n = 3146). Caption: Relative Risk (RR) of T1D incidence in relation to weekly mean temperature among individuals aged < 35-years, adjusted for sunshine hours. The reference temperature is 20 °C. Shaded area = 95% CI.

Between 2015 and 2022, 3146 patients (< 35-years) with T1D were newly diagnosed in Taiwan. Table 1 shows the patients' ages at diagnosis varied widely, with the majority (56.8%) being under 18-years-old. More males (54.5%) than females were noted in the sample. Diagnoses were distributed relatively evenly across the seasons, with a slightly lower incidence in winter (23.6%) and summer (24.2%). Diagnoses were distributed across all months, with March and November having the highest number (8.9%). Supplementary Figure S1 shows the distribution of weekly T1D cases across temperature ranges, with no clear visual trend. Similarly, Supplementary Figure S2 depicts the number of T1D cases in relation to total sunshine hours per week, showing that the number of T1D cases per week was very similar across various sunshine hours. Fig. 1 shows the Relative Risk (RR) of T1D incidence based on weekly mean daily temperature (Fig. 1 upper) and sunshine hours (Fig. 1 lower). The model adjusts for age, sex, region, season, calendar year, and month of diagnosis. Compared with the median temperature of 20 °C, the lowest and highest RRs (95% CI) for T1D were 0.65 (0.39‒1.09) at 13 °C and 1.04 (0.89‒1.25) at 23 °C, respectively. Significantly lower RRs of T1D incidence were observed across the temperature range of 17‒19 °C. Specifically, the RR and 95% CI associated with 17 °C, 18 °C, and 19 °C were 0.86 (95% CI 0.76‒0.97), 0.91 (95% CI 0.85‒0.97), and 0.95 (95% CI 0.92‒0.99), respectively, referenced to 20 °C. In contrast, no significant association was identified between weekly total sunshine hours and T1D across the range of 11‒61 hours. When both weekly mean daily temperature and total sunshine hours per week were simultaneously included in the model, the results were similar (Fig. 2).

**Table 1:** Characteristics of study patients with T1D (n = 3146).

	n	%
**Total**	3146	100.0
**Age at diagnosis (years)**		
0‒4	275	8.7
5‒9	561	17.9
10‒17	950	30.2
18‒34	1360	43.2
**Gender**		
Male	1714	54.5
Female	1432	45.5
**Season on date of diagnosis**^a^		
Spring	835	26.5
Summer	760	24.2
Fall	809	25.7
Winter	742	23.6
**Calendar year of diagnosis**		
2015	420	13.4
2016	414	13.2
2017	387	12.3
2018	375	11.9
2019	390	12.4
2020	382	12.1
2021	357	11.3
2022	421	13.4
**Month of diagnosis**		
January	260	8.3
February	224	7.1
March	279	8.9
April	278	8.8
May	278	8.8
June	249	7.9
July	245	7.8
August	266	8.5
September	253	8.0
October	276	8.8
November	280	8.9
December	258	8.2

a Spring, March‒May; Summer, June‒August; Fall, September‒November; Winter, December‒February. Std, Standard Deviation.

## Discussion

This population-based study demonstrated that neither low temperature nor shorter sunshine hours are associated with significantly higher T1D risk. While the findings of the present study do not support prior observations that lower temperature may increase the risk of T1D,^17^^,^^18^ the present study findings also did not replicate prior findings arguing that lower temperature is associated more with T1D incidence than shorter sunshine hours.^4^^,^^8^ The observed modestly reduced relative risks of T1D incidence at mean temperatures between 17° and 20 °C should be interpreted with caution. These estimates are expressed relative to the reference temperature of 20 °C and do not necessarily indicate a biologically protective effect of lower ambient temperature. Instead, this pattern likely reflects the shape of the modeled exposure-response relationship and the choice of reference point, rather than a true reduction in disease risk. Moreover, residual confounding by seasonally correlated factors such as viral circulation, behavioral patterns, or indoor environmental conditions may contribute to this finding.

Both animal experiments and epidemiological studies have found that insulin levels increase at lower temperatures, possibly to balance the rise in blood glucose levels caused by increased food intake and decreased physical activity during cold weather.^19^, ^20^, ^21^ Cold weather may also contribute to the seasonal pattern of T1D through mechanisms unrelated to metabolic overload. A well-established explanation involves increased circulation of viral pathogens during colder months, particularly enteroviruses, which have been repeatedly implicated in triggering or accelerating autoimmune destruction of pancreatic β-cells in genetically susceptible individuals.^22^, ^23^, ^24^ Viral infections can induce β-cell stress, upregulate HLA class I expression in islets, and promote autoreactive T-cell activation, providing a biologically plausible link between winter viral peaks and higher T1D incidence. In addition, cold seasons are associated with shifts in immune regulation, including enhanced expression of pro-inflammatory cytokines and reduced UV-mediated immunosuppressive signaling, which together promote a more autoreactive immunologic environment.^25^^,^^26^ Photoperiod-driven changes in melatonin secretion may further influence autoimmune pathways, as melatonin modulates both innate and adaptive immunity and has been linked to autoimmune disease activity.^27^^,^^28^ Finally, cold-induced sympathetic activation can heighten adrenergic and inflammatory signaling, potentially lowering the threshold for autoimmune responses against β-cells.^29^^,^^30^ Despite the abovementioned mechanisms providing plausible biological pathways through which low ambient temperatures may influence T1D onset in temperate climates, the present study did not find a significantly increased risk of T1D associated with lower temperature. This may be because most previous studies were conducted in temperate or cold-climate countries, whereas Taiwan is in the subtropics, where winter temperatures are not as low. As a result, the biological mechanisms proposed in earlier research may not be activated here.

This study demonstrated no significant relationship between sunshine hours and T1D incidence. Very few studies have conducted assessments on the relative importance of shorter sunshine exposure and living with lower temperatures in association with T1D incidence. A prior study in Sweden indicated that the primary factor driving the seasonal variation in T1D incidence is atmospheric temperature, not sunlight.^8^

Despite the lack of association in the subtropical setting, mechanistic studies from temperate regions suggest that vitamin D may play a role, as outlined below. Vitamin D deficiency has been implicated in the development of T1D, with studies from Norway and Denmark suggesting that reduced sunlight exposure is associated with increased T1D risk in children.^31^^,^^32^ A Norwegian nested case-control study reported lower maternal 25-OH D levels during pregnancy among T1D cases,^32^ while a Danish birth cohort found that higher maternal sunlight exposure in the third trimester was associated with reduced T1D risk in male offspring.^31^ Adequate vitamin D levels may support immune regulation and reduce autoimmune beta-cell destruction.^33^^,^^34^

Other potential mechanisms linking exposure to shorter sunshine hours to T1D involve immunomodulatory pathways. Experimental and clinical evidence suggests that adequate sunlight exposure can enhance regulatory T-cell activity, promote immune tolerance, and thereby reduce β-cell autoimmunity. In addition, Ultraviolet (UV) radiation may exert vitamin d-independent effects on the immune system: cutaneous UV exposure can alter the balance of pro- and anti-inflammatory cytokines and modulate antigen-presenting cells even in the absence of major changes in circulating 25-hydroxyvitamin D. These vitamin d-independent and UV-mediated immunomodulatory mechanisms provide biologically plausible, though still unproven, pathways through which shorter sunshine exposure could contribute to T1D development in susceptible individuals.^35^

One major strength of this study is its extension of the study area to a sub-tropical region and the study population to a non-Caucasian population. This study first simultaneously examined the potential influences of lower temperature and shorter sunshine hours. Despite the above strengths, several limitations should be noted. First, because this study identified T1D cases using only disease diagnosis codes from the NHI claims data, and clinical information for verification was not available, the possibility of T1D disease misclassification cannot be completely ruled out. Second, due to limited sample size, the authors were unable to assess the potential effect modification by age on the associations between selected meteorological factors and T1D. Third, exposure misclassification might exist in the present study because only outdoor temperature and sunshine hours were analyzed without considering the indoor environment and study subjects’ daily activities. Moreover, sunshine exposure was estimated using data from 29 meteorological stations aggregated at the regional level. Although stations were distributed across all four regions of Taiwan, their spatial distribution was not strictly proportional, with a higher concentration in western regions and fewer stations in the east. As sunshine duration can vary at a microclimatic level, particularly between northern and southern Taiwan and in mountainous areas, regional averaging may not fully capture localized exposure variability. This may have resulted in non-differential exposure misclassification, potentially biasing the estimated associations toward the null. Given the likely non-differential exposure misclassification, the authors cannot rule out the possibility that a modest true association between sunshine hours and T1D incidence exists but was attenuated toward the null. Fourth, the unit of analysis was the daily aggregate count of T1D, linked to national daily meteorological data. Within this ecological time-series design, individual-level covariates (e.g., socioeconomic status, family history of T1D, and accessibility to healthcare) could not be incorporated. In addition, residual confounding by unmeasured seasonal factors such as viral activity or air pollution cannot be excluded. Air pollution (e.g., PM_2.5_) may contribute to immune and endocrine dysregulation via adrenal changes,^36^ which could interact with low sunlight/temperature-related factors (like vitamin D deficiency) to increase the risk of type 1 diabetes. Fifth, ecological inference limits individual-level interpretation. This ecological time-series design is hypothesis-generating and cannot infer causality at the individual level.

## Conclusions

In general, the present study did not note significantly higher risk of T1D in association with lower ambient temperature or shorter sunshine hours in Taiwan. Interpretations of the study findings should be cautious due to certain methodological limitations including T1D case ascertainment, incomplete adjustment for all potential confounders, and potential exposure misclassification.

## Data availability

The NHI claim data used in this study cannot be shared due to data protection regulations enforced by the National Health Insurance Administration (NHIA) in Taiwan. Ambient temperature and sunshine hours data are available from the Central Weather Administration.

## Ethical approval

This study was approved by the Institutional Review Board of National Cheng Kung University Hospital (n°B-ER-112–034).

## Author’s contribution

Ya-Hui Chang: Conceptualization; methodology; formal analysis; writing-original draft; writing-review & editing.

Hon-Ping Ma: Conceptualization; methodology; writing-review & editing.

Hua-Fen Chen: Writing-review & editing.

Ping-Ling Chen: Writing-review & editing.

I-Lin Hsu: Writing-review & editing.

Chung-Yi Li: Conceptualization; methodology; formal analysis; writing-original draft; writing-review & editing.

## Funding

This study was supported by grants from the National Science and Technology Council (NSTC 112–2314-B-006–067-MY3), 10.13039/501100004700Taipei Medical University (TMU114-AE1-B24), and Taipei Medical University–Shuang Ho Hospital (115TMU-SHH-18).

## Conflicts of interest

The authors declare no conflicts of interest.
